# Recruitment and reach in a pragmatic behavioral weight loss randomized controlled trial: implications for real-world primary care practice

**DOI:** 10.1186/s12875-020-01117-w

**Published:** 2020-03-03

**Authors:** Christie A. Befort, Danny Kurz, Jeffrey J. VanWormer, Edward F. Ellerbeck

**Affiliations:** 1 Department of Population Health, University of Kansas Medical Center, 3901 Rainbow Blvd, MS 1008, Kansas City, KS 66160 USA; 2 Center for Clinical Epidemiology & Population Health, Marshfield Clinic Research Institute, 1000 North Oak Ave (ML2), Marshfield, WI 54449 USA

**Keywords:** Reach, Primary care, Obesity, Pragmatic randomized clinical trial

## Abstract

**Background:**

Obesity is a major risk factor behind some of the most common problems encountered in primary care. Although effective models for obesity treatment have been developed, the ‘reach’ of these interventions is poor and only a small fraction of primary care patients receive evidence-based treatment. The purpose of this study is to identify factors that impact the uptake (reach) of an evidence-based obesity treatment program within the context of a pragmatic cluster randomized controlled trial comparing three models of care delivery.

**Methods:**

Recruitment and reach were evaluated by the following measures: 1) mailing response rates, 2) referral sources among participants contacting the study team, 3) eligibility rates, 4) participation rates, and 5) representativeness based on demographics, co-morbid conditions, and healthcare utilization of 1432 enrolled participants compared to > 17,000 non-participants from the clinic-based patient populations. Referral sources and participation rates were compared across study arms and level of clinic engagement.

**Results:**

The response rate to clinic-based mailings was 13.2% and accounted for 66% of overall program recruitment. An additional 22% of recruitment came from direct clinic referrals and 11% from media, family, or friends. Of those screened, 87% were eligible; among those eligible, 86% enrolled in the trial. Participation rates did not vary across the three care delivery arms, but were higher at clinics with high compared to low provider involvement. In addition, clinics with high provider involvement had a higher rate of in clinic referrals (33% versus 16%) and a more representative sample with regards to BMI, rurality, and months since last clinic visit. However, across clinics, enrolled participants compared to non-participants were older, more likely to be female, more likely to have had a joint replacement but less likely to have CVD or smoke, and had fewer hospitalizations.

**Conclusions:**

A combination of direct patient mailings and in-clinic referrals may enhance the reach of primary care behavioral weight loss interventions, although more proactive outreach is likely necessary for men, younger patients, and those at greater medial risk. Strategies are needed to enhance provider engagement in referring patients to behavioral weight loss programs.

**Trial registration:**

clnicialtrials.gov NCT02456636. Registered May 28, 2015, https://www.clinicaltrials.gov/ct2/results?cond=&term=RE-POWER&cntry=&state=&city=&dist=.

## Background

Primary care remains an underutilized yet important resource for patients with obesity who need assistance with weight loss [1–3]. This is especially the case for patients in underserved areas such as rural or other low socioeconomic communities where access to evidence-based programs are lacking [4–6]. Although systematic reviews of obesity treatment trials in primary care settings have concluded they result in sustained modest weight loss, [7, 8] the reach of such interventions, including the proportion and representativeness of patients who participate, is largely unknown [9]. As a result, clinicians and program planners often lack information on the likelihood of achieving meaningful rates of uptake by patients, particularly patients who may have fewer resources or more competing demands [10].

Practical lessons can be learned from randomized trials that support later clinical implementation [11]. Participation rates and representativeness, however, are rarely reported despite the clear value of such information for assessing overall impact [9, 12]. One estimate has indicated that less than 30–50% of primary care trials across a variety of contexts and topics ever meet their original recruitment goals [13–15]. In a review of 19 behavioral obesity trials conducted across various community and clinical settings, only 2 reported participation rates and 1 reported on the representativeness of the study sample, none of which were conducted in primary care clinics [16].

An additional factor that limits the ability to understand the potential reach of behavioral weight loss interventions is that few of the prior trials have been highly pragmatic, whereby existing clinic staff delivered the intervention and broad eligibility criteria were used to represent real-world populations [17, 18]. Pragmatic trials are especially needed in obesity treatment, because unfortunately uptake of services under the predominant fee-for-service model has been limited [19]. Alternative care delivery models for obesity treatment, such as after-hours group visits or phone-based care, might be more acceptable to patients and expand the reach.

The aims of the current study are to evaluate patient recruitment strategies and reach within a 3-arm pragmatic cluster randomized trial comparing two alternative care delivery models to the fee-for-service model for behavioral treatment for obesity. We examine recruitment yield from different recruitment strategies, as well as eligibility and participation rates, and then compare these rates across the three study arms. We also explore recruitment strategies and participation rates across clinics that had a high versus a low level of provider involvement in recruiting patients. Finally, we examine representativeness of the trial sample by comparing demographic characteristics, co-morbid conditions, and healthcare utilization across study participants and non-participants.

## Methods

### Study design and setting

RE-POWER is a cluster randomized trial comparing three models of implementing behavioral weight loss interventions in 36 rural primary care practices in the Midwestern region of the U.S. Of the 36 randomized practices, 10 were owned by an integrated healthcare system in Wisconsin (the Marshfield Clinic), and the remaining were a mix of 13 hospital-owned clinics, 9 private practices, 3 non-profit clinics, and 1 Veteran Affairs (VA) clinic. All practices predominantly or exclusively served rural residents; 11 were designated rural health clinics and 12 were federally qualified health centers.

The study protocol has been previously reported [20]. In brief, clinics were randomized to one of three care delivery models: 1) individual face-to-face 15-min office visits modeled after the fee-for-service provision for the Centers for Medicaid and Medicare Intensive Behavior Therapy, [21] 2) 60-min group visits conducted after hours within the local practice modeled after patient-centered medical home standards (PCMH) that emphasize coordinated, comprehensive care with enhanced access, [22] and 3) 60-min group conference call visits conducted centrally modeled after a disease management approach (DM). For fee-for-service and PCMH arms, all sessions were provided by clinic-employed providers (e.g., primary care provider [PCP], registered nurse [RN], registered dietitian [RD], licensed clinical social worker [LCSW]). Session frequency started as weekly and tapered to monthly by 6 months and remained monthly through 24 months. The intervention across all three study arms included evidence-based behavioral components for reducing caloric intake, increasing physical activity, daily self-monitoring, and goal-setting. Treatment goals were 5 to 10% weight loss during the first 6 months, followed by weight loss maintenance from 6 to 24 months. The study was approved by a central Institutional Review Board [23] and the VA Nebraska-Western Iowa IRB. Recruitment and reach data were obtained from mailing lists, participant report of how they were referred to the study, screening outcomes, and enrollment outcomes. These data were collected from January 2016 to October 2017; analyses were conducted in 2019.

### Eligibility criteria

As a pragmatic study, the exclusion criteria were kept to a minimum. Patients were eligible if they were between 20 and 75 years-old, had a body mass index (BMI) between 30 and 45 kg/m^2^, and resided in a rural location [24]. Medical clearance from the treating PCP was required, and patients must have been seen in the clinic within the past 18 months. Exclusions included a history of bariatric surgery or planned bariatric surgery within 2 years, pregnancy in the last 6 months, currently lactating, myocardial infarction, stroke, or new cancer diagnosis in the last 6 months, or plans to change primary care clinics during the study. One individual per household was allowed to enroll.

### Recruitment strategies

The two primary recruitment strategies were direct mailings and in-clinic referrals. Each practice was responsible for developing a mailing list of potentially eligible patients, based on eligibility criteria of age, obesity status, rural zip code, and a clinic visit within the past 18 months. Invitation letters signed by the local PCP and study principal investigator were mailed to patients, along with a study brochure and a pre-stamped opt-in postcard. Separate recruitment materials were designed for each study arm to reflect the nature of the three intervention delivery models. Mailings were sent in waves for each clinic until the recruitment target was met or the entire list was exhausted. Providers also referred patients during routine medical visits, and study brochures and opt-in postcards were distributed in clinics. Fourteen of the 36 clinics also elected to use advertisements placed around the community, printed in local newspapers, and/or posted on clinic webpages and social media.

### Patient screening

Interested patients opted in for screening by contacting the central study team by phone, e-mail, or the opt-in postcard. Patients who responded were asked how they first heard about the study. If eligible by phone screening, PCP clearance was obtained, and patients were sent a baseline survey. After completion of the survey, local clinic staff called the patient to schedule a baseline visit at which time they verified BMI, obtained informed consent, and collected baseline lab measures.

### Measures

#### Recruitment sources and mailing response rate

We report the total number and percent of patients who contacted the study team across different recruitment sources (self-reported by patients), as well as the median and range per clinic within each of the three randomization arms. The response rate to the mailing is calculated as the number of patients who contacted the study who received the mailing divided by the total number of letters mailed (excluding the number of undeliverable mailings).

#### Participation rates

Among patients who were screened, we report the number and percent who were eligible and who declined, in total and by study arm. We report different participation rates as recommended by Glasgow [25]. From least to most conservative, participation rates include the number enrolled divided by 1) the number who screened and were assumed eligible (applying the eligibility rate at each screening step to those who declined prior to completing screening), 2) the number who contacted the study line and assumed eligible, and 3) the number mailed to and assumed eligible.

#### Provider involvement (low versus high) in creating recruitment lists

The level of provider input in curating the recruitment list varied across the sites, from a completely hands-off population-based approach (electronic medical record (EMR)-extracted list with no provider review) to a highly involved approach (list reviewed for medical and behavioral contraindications and/or some patients prioritized to receive the mailing first). These differences arose by natural variation during the conduct of the trial. Therefore, we explored whether recruitment sources, eligibility rates, and representativeness of enrolled participants varied across level of provider involvement. We created two categories of clinics (low verses high involvement) by reviewing detailed descriptions of how the lists were created along with the number of patients they excluded and/or prioritized. Low involvement clinics (*n* = 24) excluded few of the patients originally identified (median = 0%, range 0–16%) and prioritized very few to receive the mailing first (median = 0%, range 0–4%). High involvement clinics (*n* = 12) excluded a higher proportion of patients (median = 6%, range 0–39%) and/or prioritized a higher number of patients to receive the mailing first (median 28%, range 0–69%).

#### Characteristics of participants versus non-participants

We compare age, sex, race, rurality (Rural-Urban Commuting Area Codes), [24] months since last clinic visit, and BMI (kg/m^2^) between enrolled participants and non-participants who were patients at the participating clinics. We also explore these characteristics for participants enrolled from clinics with high versus low level of provider involvement.

For the 10 Marshfield Clinic sites, additional characteristics were available from EMR extraction for all patients who met the initial eligibility criteria of age, BMI, rural residence, clinic visit within 18 months, and were medically-homed to the Marshfield Clinic Health System with reasonably complete capture of all medical care, per standard quality reporting definitions used by the Marshfield Clinic Institute for Quality, Innovation and Patient Safety [26]. Additional extracted variables included co-morbid conditions, smoking status, number of ambulatory visits, and in-patient days over the past 3 years.

### Analyses

Analysis of variance was conducted to examine differences across study arms in mailing response rates, ineligible and decline rates, and participation rates. Rates were calculated at the clinic-level and then averaged across clinics to account for site level variation. T-tests were used to explore differences across clinics with high versus low level of involvement. To evaluate representativeness, characteristics of study participants versus non-participants were compared at the clinic-level using paired t-tests. For demographic and BMI characteristics, non-participants include 13,858 patients who were mailed the study invitation, 3539 who were on the mailing lists but did not receive the mailing due the recruitment goal being met, and 100 who were patients at a participating clinic and called the study line but were not on the mailing list. To examine the potential impact of non-uniform methods across clinics in creating the mailing lists, two sensitivity analyses were conducted. First, we compared characteristics across enrolled participants to only non-participants who received the study mailing. Second, we compared characteristics just among the enrolled and non-enrolled sample at the 10 Marshfield Clinic sites, where uniform population-based EMR data extraction methods were used to create the lists. Finally, we also compared results with and without the single VA site, because their recruitment process varied and was handled locally due to VA IRB restrictions. Results were the same, thus findings are presented with this site included. All analyses were conducted with SPSS version 25.

## Results

### Mailing response

Mailings were sent to 15,076 patients with a median of 357 mailings per clinic (Additional file 1: Figure S1). Of these, 1990 patients contacted the study line, for mailing response rate of 13.2%. Of these contacts, only 1383 self-reported they heard about the study from the mailing while the remainder reported they heard about the study during a clinic visit (*n* =247) or from media or family/friends (*n* = 67). There was no difference in mailing response rates by study arms (*p* = 0.42).

### Recruitment sources

Of 2479 potential participants contacting the study, 66.1% reported being referred by the mailing, 21.5% by a provider during a clinic visit, and 11.0% from media or family/friends (Table 1). When restricting these analyses to just the sample who ultimately enrolled in the study (*n* = 1432), self-reported referral sources were similar (see supplemental Table S1). Clinics randomized to the DM arm had a higher proportion of patients recruited through media or family/friends (17.5% versus 6.1% for fee-for-service and 8.8% PCMH); *p* = 0.02.

**Table 1:** Percent of patients from different recruitment sources, among patients who contacted the study (*n* = 2479)

	In-clinic individual intervention (FFS) 12 clinics	In-clinic group intervention (PCMH) 12 clinics	Phone group intervention (DM) 12 clinics		Total 36 clinics
	Mean (SD)	Mean (SD)	Mean (SD)	***p***	Mean (SD)
Mailings	65.8% (27.8)	67.6% (11.7)	64.7% (18.9)	0.94	66.1% (20.0)
In clinic referral	26.6% (26.7)	21.1% (10.3)	17.0% (10.0)	0.41	21.5% (17.4)
Other sources	6.4% (5.1)	8.9% (8.2)	17.7% (14.5)	0.02	11.0% (10.9)

Data are reported as means at the clinic level. Thirty-three contacts missing recruitment source

Compared to clinics categorized as low involvement, high involvement clinics had a greater proportion of contacts from in-clinic referrals (32.8% versus 15.9%, respectively; *p* = 0.004) and a lower proportion from the mailing (55.8% versus 71.2%; *p* = 0.027); see supplemental Table S2. However, there was a high degree of clinic-level variation in the proportion of contacts from in-clinic referrals, ranging from 5.4 to 97.6% among high involvement clinics, and 0 to 29.8% among low involvement clinics.

### Eligibility and participation rates

**Fig. 1:**
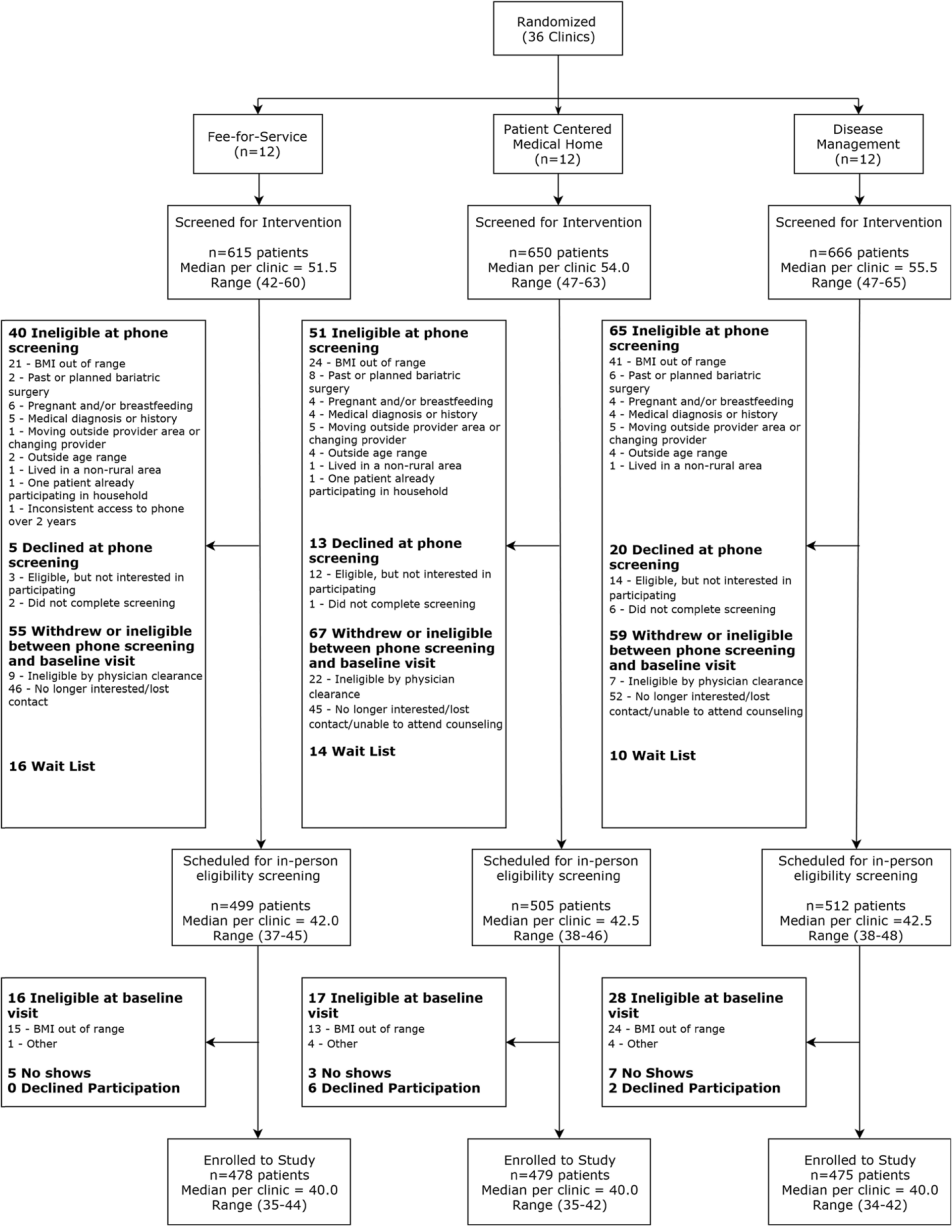
Participant flow from screening to enrollment

Figure 1 shows participant flow from screening to enrollment, and Table 2 summarizes the number and percent of screened participants who were ineligible and declined at various stages. There were no differences in ineligible or decline rates across study arms, or in participation rates. Of those screened, 13.2% were ineligible overall (86.8% eligibility rate) with 8.1% ineligible at phone screening. The top reason for ineligibility at phone screening was BMI out of range (Fig. 1). Only 2% (*n* = 38) of those who screened for the study were not cleared to participate by their PCP. Only 3% (*n* = 61) were ineligible at the baseline visit, with the vast majority of these (*n* = 52) due to verified BMI being out of range (Fig. 1).

**Table 2:** Number of patients who were ineligible or declined to participate, as percent of patients screened (*n* = 1931), by study arm

	In-clinic Individual Intervention (FFS)	In-clinic Group Intervention (PCMH)	Phone Group Intervention (DM)	***p***	Total
**Screened total**	**615**	**650**	**666**		**1931**
**Ineligible total**	**65 (10.6%)**	**90 (13.8%)**	**100 (15.0%)**	0.14	**255 (13.2%)**
at phone screening	40	51	65		156 (8.1%)
by physician clearance	9	22	7		38 (2.0%)
at baseline visit	16	17	28		61 (3.2%)
**Declined total**	**56 (9.1%)**	**67 (10.3%)**	**81 (12.1%)**	0.24	**204 (10.6%)**
at phone screening	5	13	20		38 (2.0%)
between phone screening and baseline visit	46	45	52		143 (7.4%)
at baseline visit/no show	5	9	9		23 (1.2%)

Overall 10.6% of those who screened declined to participate, and the majority of these declined after the phone screening but prior to their baseline/consent visit. The number of participants who no showed (*n* = 15) or declined (*n* = 8) at the baseline/consent visit was very low. The clinic-level participation rates were as follows: 86.0% of those who screened eligible, 2) 66.3% of those who contacted the study line and presumed eligible, and 3) 15.7% of those who received the study mailing and presumed eligible.

Compared to clinics categorized as low involvement, patients from high involvement clinics had a lower ineligible rate (9.4% versus 14.9%, respectively; *p* = 0.005) as well as a lower decline rate (7.2% versus 12.1%, respectively; *p* = 0.003); see supplemental Table S3. Likewise, high involvement clinics had higher participation rates compared to low involvement clinics: 89.8% vs. 84.3% of those screened eligible, 78.1% vs. 62.4% of those who contacted the study line and presumed eligible, and 23.4% vs. 12.2% of those who received the study mailing and presumed eligible; all *p*’s < 0.01.

### Comparison of participants and non-participants

Table 3 shows select characteristics of enrolled participants versus non-participants. Compared to non-participants, enrolled participants were significantly older (54.1 versus 51.3 years-old; *p* < 0.001), had a higher BMI (36.5 versus 35.6 kg/m^2^; *p* < 0.001), and were seen more recently in the clinic (3.9 versus 4.7 months since last visit; *p* = 0.02). They also were more likely to be female (76.9% versus 55.0%; *p* < 0.001) and to live in an isolated rural area (46.3% versus 41.7%; *p* < 0.001). In sensitivity analyses to examine the potential influence of the mailing on representativeness, comparisons on these characteristics were also made between enrolled participants and only those non-participants who were mailed a study invitation (*n* = 13,858), as well as for the subsample of participants and non-participants from the 10 Marshfield Clinic sites. In both of these sensitivity analyses, the results were the same with only one exception: there were no differences in rurality between participants and non-participants at the Marshfield sites.

**Table 3:** Demographics and BMI of enrolled participants versus non-participants who were patients at participating clinics

	Enrolled participants (*n* = 1432, 36 clinics)	Non-participants (***n*** = 17,497, 36 clinics)^**a**^	***p*** value
	Mean (SD)	Mean (SD)	
Age	54.1 (4.1)	51.3 (4.3)	**< 0.001**
BMI (kg/m^2^) from registry	36.5 (0.7)	35.6 (1.2)	**< 0.001**
Sex			
Female %	76.9% (18.0)	55.0% (15.6)	**< 0.001**
Race/Ethnicity			
White non-Hispanic %	95.8% (4.9)	94.3% (10.2)	0.26
White Hispanic %	1.7% (2.6)	3.1% (8.7)	0.23
Other %	2.5% (3.4)	2.7% (2.6)	0.79
Rurality			
Large %	35.9% (38.3)	37.7% (37.8)	0.05
Small %	17.8% (27.8)	20.6% (28.0)	**0.03**
Isolated %	46.3% (36.5)	41.7% (34.4)	**0.001**
Months since last clinic visit	3.9 (1.4)	4.7 (1.8)	**0.02**

Data are reported as means at the clinic level

^a^Missing data for non-participants from full sample varies based on variables included in each practice list; *n* = 16,794 for age, 17,420 for sex, 15,808 for race/ethnicity, 17,497 for rurality, 15,678 for months since last visit, and 15,747 for BMI

Table 4 shows co-morbid conditions and healthcare utilization for the subsample of participants and non-participants from Marshfield Clinic sites. Compared to non-participants, enrolled participants were less likely to have cardiovascular disease (2.5% versus 5.0%; *p* = 0.03) and to currently smoke (5.9% versus 16.9%; *p* < 0.001) but more likely to have prior joint replacement surgery (10.9% versus 8.1%; *p* = 0.02). They also had fewer inpatient days over the previous 3 years; *p* = 0.001. No differences were observed for type 2 diabetes, history of cancer, kidney disease, pulmonary disease, depression, or number of outpatient encounters.

**Table 4:** Medical co-morbidities and healthcare utilization of participants versus non-participants who were patients at a subset of clinics^b^

	Enrolled participants (***n*** = 402; 10 clinics)	Non-participants (***n*** = 6199; 10 clinics)	
**Comorbid conditions**			
Type 2 Diabetes	16.9% (7.6%)	14.1% (4.2)	0.14
Cardiovascular disease	2.5% (2.4)	5.0% (2.2)	**0.03**
Cancer	21.2% (7.2)	17.0% (3.5)	0.11
Chronic kidney disease	7.5% (3.5)	7.9% (2.7)	0.71
Chronic pulmonary disease	13.7% (4.8)	14.4% (1.8)	0.65
Joint replacement history	10.9% (4.4)	8.1% (2.7)	**0.02**
Depression	7.0% (4.9)	7.4% (3.1)	0.84
Current smoker	5.9% (4.1)	16.9% (5.3)	**< 0.001**
Former Smoker	29.2% (7.6)	27.8% (7.1)	0.58
**Ambulatory visits in past 3 years**	13.9 (2.6)	13.0 (1.0)	0.32
**Inpatient days in past 3 years**	0.41 (0.245)	.98 (0.37)	**0.001**

Data are reported as means at the clinic level

^b^The subset of clinics includes 10 sites within the Marshfield Clinic healthcare system

Finally, we explored the impact of provider involvement on representativeness of the sample by comparing enrolled participants versus non-participants separately within high versus low involvement clinics (see supplemental Tables S4 and S5). Within both high and low involvement clinics, enrolled participants were significantly older (*p*’s = 0.006 and < 0.001, respectively) and more likely to be female (*p*’s < 0.001). However, only within the low involvement clinics were enrolled participants significantly more likely to have a higher BMI (*p* < 0.001), to be seen in the clinic more recently (*p* = 0.005), and to live in a more isolated area (*p* = 0.005); within the high involvement clinics, differences on these characteristics between participants and non-participants were non-significant.

## Discussion

This study examined recruitment strategies and patient reach for a pragmatic trial comparing three models of primary care delivery for implementing behavioral therapy for obesity. The main recruitment strategy was direct mailings to patients who appeared eligible based on chart review of age, BMI, and rurality criteria. The response rate to the mailing was 13.2%, i.e. for every 30 individuals mailed an invitation, 4 responded. This rate is very similar to the 13.6% mailing response rate we observed in a prior obesity treatment trial among rural breast cancer survivors identified through local cancer registries, [27] and substantially higher than < 1% response rates seen in untargeted mailings to a general population [28, 29].

Perhaps most encouraging, the response to recruitment strategies and participation rates were consistent across clinics randomized to the three different study arms. Although these findings need confirmation in future studies, they suggest that the different models of delivering the weight management sessions (e.g., at the local clinic versus over the phone, in a group versus individual setting) were about equally ‘attractive’ to invited individuals. This may assuage some concerns around program format, as for example, the travel requirements for in-person delivery did not appear to present a systematically greater enrollment barrier. Factors such as the level of provider encouragement and patient readiness could be more critical for patients’ decisions to engage in a health promotion program [30].

The success of PCP referrals in recruiting patients into primary care obesity treatment trials varies widely across studies, from being completely ineffective [31] to being the sole recruitment strategy [32]. This reveals a highly variable level of engagement of providers in the practical conduct of research-based interventions. We observed this level of variation even among the clinics participating in this trial, from highly engaged PCPs where 98% of participants came from in-clinic referrals, to very low engagement where none came from provider referrals. Comparisons across clinics categorized as high versus low level of provider involvement showed that higher involvement doubled the proportion of patients from in-clinic referrals (33% versus 16%). Ngune et al. has highlighted the importance of bona fide engagement with PCPs to ensure that research is clinically relevant and useful, rather than relying soley on practice databases to access patients [33]. Without this necessary engagement and support, providers often report barriers such as forgetting to refer patients or assuming patients are uninterested [34, 35]. As evidenced by the within study variability we observed in RE-POWER, approaches to accomplishing provider engagement need to be tailored to each unique clinic-level context [36].

The majority of prior primary care obesity treatment trials have recruited primarily through proactive phone calls following an introductory mailing, rather than relying on patients to make the first contact [32, 37–42]. Recruitment strategies can be classified on a continuum of passive (general advertisements) to active (calling patients already identified as eligible after thorough chart review). The approach we used, i.e. a combination of targeted mailings, provider referrals, and clinic-driven advertisements), falls in the middle of this continuum. Passive strategies are typically less costly but also have lower yield. In contrast, proactive phone calls are more time-consuming and less applicable in clinical practice, [43] but may reach certain populations better. For example, one study found that participants recruited through proactive phone calls, compared to participants who made the first contact, were more likely to be racial/ethnic minorities and to have lower education [25]. A cost analysis of recruitment methods for clinic-based pediatric obesity trials found that provider referrals was the most cost-effective, and the combination of provider referrals with targeted mailings the most successful [44]. Our findings support the combined strategy of targeted mailings and provider referrals. Only 11% of patient contacts came from passive recruitment methods of either media or word-of-mouth referrals. The low rate of word-of-mouth referrals is notable for the rural setting of this trial, where one might expect greater family/friend referrals in small communities.

It is important to distinguish between eligibility rates, which is a function of the study-specific inclusion/exclusion criteria driven by internal validity concerns, and participation rates which represent uptake by patients meeting the eligibility criteria. As expected due to our pragmatic study design with limited eligibility criteria, we observed a high eligibility rate (87%) in comparison to previous primary care-based weight loss trials which noted eligibility rates ranging from 21 to 66% [32, 37–39, 42, 43, 45, 46]. Participation rates are difficult to compare to other studies due to a sparse literature and inconsistency in how they are calculated. We observed participation rates of 86% of those who screened eligible and 16% of those who received the mailing. An internet-based weight loss trial that used targeted mailings to health plan members observed a 5% participation rate [47]. To enable comparison to a larger group of studies, we estimated participation rates in prior primary care behavioral weight loss trials from published participant flow diagrams where possible. These studies recruited through proactive phone calls, and using as the denominator the number of patients for whom contact was attempted multiplied by the eligibility rate, participation rates ranged from 26 to 61% of patients called [25, 37–40]. Although proactive phone calls may result in higher participation rates, due to added cost and clinical burden, this strategy may be best reserved for subgroups who may be less likely to respond to mailings.

Our analysis of representativeness is one of the first to empirically compare characteristics of participants to the broader primary care clinical populations. Overall, we found participants to be older and more likely to be female. Within our subsample of Marshfield Clinic sites, participants were also less likely to have cardiovascular disease, to smoke, and had fewer inpatient hospital days. In addition, within the low involvement clinics (but not the high involvement clinics), participants were more likely to have a higher BMI and to live in a more isolated rural area. This latter finding appears to be driven by the clinics that were located in isolated areas, where clinics with low involvement were less likely to draw from larger surrounding areas compared to clinics with high involvement.

Representativeness is rarely reported in weight loss trials, but our findings are largely consistent with a previous trial in 3 health maintenance organization (HMO) settings where enrollees were more likely to be female, non-smokers, and had a lower disease risk score [47]. There is a long history of men being under-represented in behavioral weight loss trials, [48, 49] and surface-level tailoring of recruitment materials, e.g. including pictures of men as we did in this study, is likely insufficient. Our findings also suggest that provider engagement alone may be inadequate to recruit representative numbers of men and younger patients. More proactive recruitment strategies, including explicit training for providers to raise awareness and address any communication concerns in referring men and younger patients, may be needed.

This study has several limitations. First, the comparison of different recruitment sources is based on patient self-report of how they first heard about the study, and many who reported being referred during a clinic visit also received the mailing. We are unable to examine the impact of receiving information about the study from multiple sources, and it could be that a particular combination of referral sources has the greatest effect. Second, participants are all rural residents, and the cultural and geographical context of the trial may have had a bearing on the findings. Finally, in our comparison of participants and non-participants, we are unable to fully account for potential provider selection biases in creating the patient lists. Despite original intentions for how lists would be created, in reality the process had to be variable across sites to accommodate system capabilities and preferences. However, our sensitivity analysis with just the Marshfield Clinic sites that had uniform EMR-based curation of lists resulted in the same findings. The major strengths of this study include the unique analyses of representativeness, the large sample and diverse group of practice settings, and the pragmatic design of the trial (including the broad eligibility criteria and intervention delivery by clinic-employed staff) all of which allow for greater application of the findings to clinical practice.

## Conclusions

In conclusion, findings support the use of direct patient mailings and in-clinic referrals for recruiting into a primary care behavioral weight loss intervention, although more proactive outreach is likely necessary for men, younger patients, and those at greater medial risk. Level of provider involvement had greater influence on participation rates than type of intervention sessions. High provider involvement, compared to low involvement, also resulted in a more representative sample for some characteristics (BMI, rurality, and months since last clinic visit), but not for gender or age. Further research is needed to develop strategies for enhancing provider engagement in referring patients to behavioral weight loss programs, particularly men and younger patients.

## Supplementary information

**Additional file 1:Table S1.** Percent of patients from different recruitment sources, among patients who enrolled in the study (*n* = 1432), by study arm. **Table S2.** Percent of patients from different recruitment sources among patients who contacted study team (*n* = 2479), by clinics with high versus low level of involvement. **Table S3**. Number of patients who were ineligible or declined to participate, as percent of patients screened (*n* = 1931), by clinics with high versus low level of involvement. **Table S4.** Demographics and BMI of enrolled participants versus non-participants from clinics with high provider involvement. **Table S5.** Demographics and BMI of enrolled participants versus non-participants from clinics with low provider involvement. **Figure S1.** Referral source from 2479 patients who contacted study team. Note: See Fig. 1. for participant flow subsequent to phone screening.

## Data Availability

The datasets used and/or analysed during the current study are available from the corresponding author on reasonable request.

## Abbreviations

BMI: Body mass index
DM: Disease management
EMR: Electronic medical record
FFS: Fee-for-service
HMO: Health maintenance organization
LCSW: Licensed clinical social worker
PCP: Primary care provider
PMCH: Patient centered medical home
RD: Registered dietitian
RN: Registered nurse
