# Supplementary material for: Recruitment and reach in a pragmatic behavioral weight loss randomized controlled trial: implications for real-world primary care practice
Source: BMC Fam Pract. 2020 Mar 3;21:47. doi: 10.1186/s12875-020-01117-w (PMC7055122; doi:10.1186/s12875-020-01117-w)
Supplement: Supplementary file 1 — Additional file 1:Table S1. Percent of patients from different recruitment sources, among patients who enrolled in the study (n = 1432), by study arm. Table S2. Percent of patients from different recruitment sources among patients who contacted study team (n = 2479), by clinics with high versus low level of involvement. Table S3. Number of patients who were ineligible or declined to participate, as percent of patients screened (n = 1931), by clinics with high versus low level of involvement. Table S4. Demographics and BMI of enrolled participants versus non-participants from clinics with high provider involvement. Table S5. Demographics and BMI of enrolled participants versus non-participants from clinics with low provider involvement. Figure S1. Referral source from 2479 patients who contacted study team. Note: See Fig. 1. for participant flow subsequent to phone screening. [file 12875_2020_1117_MOESM1_ESM.docx]

**Table S1.** Percent of patients from different recruitment sources, among patients who enrolled in the study (n = 1432), by study arm

|  | **In-clinic Individual Intervention**  **(FFS)**  **12 clinics** | **In-clinic Group Intervention**  **(PCMH)**  **12 clinics** | **Phone Group Intervention**  **(DM)**  **12 clinics** |  | **Total**  **36 clinics** |
| --- | --- | --- | --- | --- | --- |
|  | **Mean (SD)** | **Mean (SD)** | **Mean (SD)** | ***p*** | **Mean (SD)** |
| Mailings | 62.8% (28.4) | 64.6% (16.4) | 60.4% (19.2) | 0.90 | 66.1% (20.0) |
| In clinic referral | 29.8% (27.0) | 26.9% (14.2) | 21.0% (10.8) | 0.51 | 21.5% (17.4) |
| Other sources | 7.4% (6.3) | 8.6% (7.6) | 18.6% (14.8) | 0.02 | 11.0% (10.9) |

**Table S2.** Percent of patients from different recruitment sources among patients who contacted study team (n = 2479), by clinics with high versus low level of involvement

|  | **High Involvement**  **12 clinics** | **Low Involvement**  **24 clinics** |  |
| --- | --- | --- | --- |
|  | **Mean (SD)** | **Mean (SD)** | ***p*** |
| Mailings | 55.8% (22.0) | 71.2% (17.2) | **0.03** |
| In clinic referral | 32.8% (24.6) | 15.9% (8.6) | **<0.01** |
| Other sources | 9.0% (8.2) | 12.0% (12.1) | 0.43 |

**Table S3**. Number of patients who were ineligible or declined to participate, as percent of patients screened (n = 1931), by clinics with high versus low level of involvement

|  | **High Involvement**  **12 clinics** | **Low Involvement**  **24 clinics** | ***p*** |
| --- | --- | --- | --- |
| **Screened total** | **596** | **1335** |  |
| **Ineligible total** | **56 (9.4%)** | **199 (14.9%)** | **0.005** |
| at phone screening | 28 | 128 |  |
| by physician clearance | 11 | 27 |  |
| at baseline visit | 17 | 44 |  |
| **Declined total** | **43 (7.2%)** | **161 (12.1%)** | **0.003** |
| at phone screening | 4 | 34 |  |
| between phone screening and baseline visit | 30 | 113 |  |
| at baseline visit/no show | 9 | 14 |  |

**Table S4.** Demographics and BMI of enrolled participants versus non-participants from clinics with high provider involvement

|  | **Enrolled participants**  **(n = 486, 12 clinics)** | **Non-participants**  **(n = 3519, 12 clinics)^a^** |  |
| --- | --- | --- | --- |
|  | **Mean (SD)** | **Mean (SD)** | ***p* value** |
| Age | 53.9 (3.7) | 51.1 (3.2) | **0.006** |
| Sex |  |  |  |
| Female % | 73.0% (23.9) | 51.9% (20.1) | **<0.001** |
| Race/Ethnicity |  |  |  |
| White non-Hispanic % | 96.9% (2.1) | 98.2% (1.6) | 0.064 |
| White Hispanic % | 1.4% (1.9) | 0.5% (.8) | 0.139 |
| Other % | 1.7% (1.2) | 1.3% (1.3) | 0.510 |
| Rurality |  |  |  |
| Large % | 27.1% (36.4) | 28.1% (37.2) | 0.526 |
| Small % | 19.4% (30.0) | 22.7% (33.7) | 0.184 |
| Isolated % | 53.4% (40.4) | 49.2% (41.0) | 0.085 |
| Months since last clinic visit | 4.3 (1.5) | 4.6 (1.9) | 0.745 |
| BMI (kg/m^2^) from registry | 36.4 (0.6) | 36.3 (1.5) | 0.954 |
| ^a^Missing data for non-participants varies based on variables included in each practice list; n = 3343 for age, 3991 for sex, 2772 for race/ethnicity, 4005 for rurality, 2866 for months since last visit, and 2952 for BMI. | | | |

**Table S5.** Demographics and BMI of enrolled participants versus non-participants from clinics with low provider involvement

|  | **Enrolled participants**  **(n = 946, 24 clinics)** | **Non-participants**  **(n = 13978, 24 clinics)^a^** |  |
| --- | --- | --- | --- |
|  | **Mean (SD)** | **Mean (SD)** | ***p* value** |
| Age | 54.2 (4.4) | 51.4 (4.9) | **<0.001** |
| Sex |  |  |  |
| Female % | 78.9% (14.4) | 56.5% (13.0) | **0.001** |
| Race/Ethnicity |  |  |  |
| White non-Hispanic % | 95.3% (5.8) | 92.3% (12.0) | 0.142 |
| White Hispanic % | 1.8% (2.9) | 4.4% (10.5) | 0.141 |
| Other % | 3.0% (4.0) | 3.3% (2.8) | 0.585 |
| Rurality |  |  |  |
| Large % | 40.3% (39.2) | 42.5% (37.9) | 0.062 |
| Small % | 17.0% (27.3) | 19.6% (25.4) | 0.101 |
| Isolated % | 42.7% (34.8) | 37.9% (30.9) | **0.006** |
| Months since last clinic visit | 3.8 (1.4) | 4.7 (1.9) | **0.005** |
| BMI (kg/m^2^) from registry | 36.6 (0.7) | 35.2 (0.8) | **<0.001** |
| ^a^ Missing data for non-participants varies based on variables included in each practice list; n = 14883 for age, 14861 for sex, 14468 for race/ethnicity, 14924 for rurality, 14149 for months since last visit, and 14227 for BMI. | | | |

**Fig S1.** Referral source from 2479 patients who contacted study team.

Note: See Fig 1. for participant flow subsequent to phone screening.

Proceeded to phone screening

N = 1931

Contacts from unknown referral source

N = 33

Contacts from media, family/friend referrals

N = 275 (67 also received mailing)

Contacts from in-clinic referrals

N = 495

(247 also received mailing)

Contacts from those

mailed to

N = 1990

(1383 self-reported mailing as referral source)

Recruitment mailings sent

N = 15,076

(Median per clinic = 357)
